# Fluorescence Spectroscopy of Porphyrins and Phthalocyanines: Some Insights into Supramolecular Self-Assembly, Microencapsulation, and Imaging Microscopy

**DOI:** 10.3390/molecules26144264

**Published:** 2021-07-14

**Authors:** Raquel Teixeira, Vanda Vaz Serra, David Botequim, Pedro M. R. Paulo, Suzana M. Andrade, Sílvia M. B. Costa

**Affiliations:** Centro de Química Estrutural, Instituto Superior Técnico, Universidade de Lisboa, Av. Rovisco Pais 1, 1049-001 Lisboa, Portugal or raquel.teixeira@ips.pt (R.T.); vanda.serra@tecnico.ulisboa.pt (V.V.S.); davidbotequim@tecnico.ulisboa.pt (D.B.)

**Keywords:** porphyrins and phthalocyanines, supramolecular self-assembly, microencapsulation, fluorescence microscopy

## Abstract

The molecular interactions of anionic tetrasulfonate phenyl porphyrin (TPPS) with poly(amido amine) (PAMAM) dendrimers of generation 2.0 and 4.0 (G2 and G4, respectively) forming H- or J-aggregates, as well as with human and bovine serum albumin proteins (HSA and BSA), were reviewed in the context of self-assembly molecular complementarity. The spectroscopic studies were extended to the association of aluminum phthtalocyanine (AlPCS_4_) detected with a PAMAM G4 dendrimer with fluorescence studies in both steady state and dynamic state, as well as due to the fluorescence quenching associated to electron-transfer with a distribution of lifetimes. The functionalization of TPPS with peripheral substituents enables the assignment of spontaneous pH-induced aggregates with different and well-defined morphologies. Other work reported in the literature, in particular with soft self-assembly materials, fall in the same area with particular interest for the environment. The microencapsulation of TPPS studies into polyelectrolyte capsules was developed quite recently and aroused much interest, which is well supported and complemented by the extensive data reported on the Imaging Microscopy section of the Luminescence of Porphyrins and Phthalocyanines included in the present review.

## 1. Introduction

The phenomenon of photosynthesis starts with the absorption of light by a chromophore molecule or a set of chromophore molecules in a light-harvesting antenna consisting of many chromophores collecting light energy, which is delivered to a reaction center where charge separation occurs leading to the storage of chemical energy [1,2,3,4,5]. Chromophores most widely used for light-harvesting in nature are chlorophylls and bacteriochlorophylls, which are macrocyclic tetrapyrrole compounds.

Porphyrins and their synthetic analogues are also largely used chromophores in artificial light-harvesting systems [6,7]. Therefore, porphyrins and related tetrapyrroles have been extensively studied due to their importance in biological processes. These related classes of molecules play a major role in the biochemistry of all living systems, mainly in biological redox processes such as photosynthesis and respiration, as well as in many other enzymatic reactions. They are often used in the development of artificial photosynthesis or light-harvesting systems, catalysis, and sensor systems [8]. The porphyrin ring has an extended conjugated π-electron system and aromatic character, which makes these compounds strong light-absorbing molecules in nature, and therefore they are often referred to as “the Pigments of Life”.

## 2. Supramolecular Self-Assembly

Concepts from supramolecular and molecular chemistry have been linked by Lehn, J.M. [9] in the so-called constitutional dynamic chemistry (CDC), which is relevant for dynamic aspects such as reversible bonds, self-organization, molecular recognition, and donor–acceptor interactions [10]. CDC is promising for nanoscience and nanotechnology since new properties and reactivity from functional supramolecular architectures can be found and rationalized. Supramolecular chemistry has been conceived as the chemistry governed by the association of two or more species interacting non-covalently to produce highly complex structures. The chemistry of the intermolecular bond formed by the association of two or more chemical species constitutes the field of supramolecular chemistry [11]. The principles of molecular complementarity form the basis of molecular recognition in the binding of the receptor-substrate. This can be found in spherical and tetrahedral recognition, metallo-receptors, amphiphilic receptors, and anion coordination [12,13]. Molecular Self-Assembly (MSA) is the process through which molecules spontaneously adopt a defined arrangement without interference from external biological systems. Self-organization is a non-equilibrium process whereas self-assembly is a spontaneous process that leads toward equilibrium. Static self-assembly requires components to remain essentially unchanged throughout the process, whereas in dynamic self-assembly, pre-existing patterns organized by specific local interactions are commonly described as self-organized [12,13]. Besides the thermodynamic differences between the two, there is also a difference as regards the minimum number of units to make an ordered material. Self-organization appears to have a minimum number of units whereas self-assembly does not necessarily imply this requisite.

Biomimetic porphyrins and chlorophylls contain specific shapes and sizes, which facilitates intermolecular interactions and therefore the formation of supramolecular interactions can be expected [14]. Functional building blocks may also assemble molecules of porphyrin, but these structures will not retain the intrinsic and emergent properties achieved by self-assembly. The supramolecular organization provides a simple mechanism to assemble a great number of molecules with structures linking nanometers to macroscopic dimensions, therefore covering several length scales [15,16,17].

The next section of this article covers several works dealing with non-covalent self-assembled interactions in the excited state detected by static and dynamic luminescence techniques.

### 2.1. Luminescence of Porphyrins and Phthalocyanines Dyes

#### 2.1.1. Porphyrinoids’ Self-Assembly by Different Templates 

**Figure 1:**
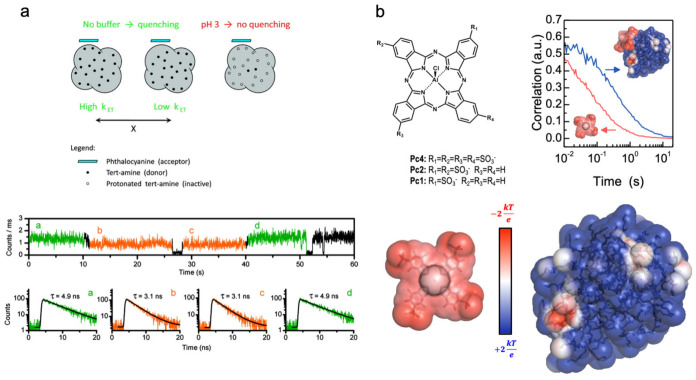
(**a**) Scheme of phthalocyanine-dendrimer complex (top) highlighting the tetrapyrrolic macrocycle (cyan) at the dendrimer surface (grey). The phthalocyanine’s fluorescence is quenched by the dendrimer’s tertiary amine donors (closed dots) except when protonated at low pH (open dots) in an aqueous medium or in hydrophilic films. Example of a single-molecule fluorescence trace of phthalocyanine-dendrimer emission (bottom) showing concomitant changes in intensity and lifetime attributed to dynamic heterogeneity in photoinduced electron-transfer due to fluctuations in donor–acceptor distance. Adapted with permission from [21]. Copyright 2010 American Chemical Society. (**b**) Chemical structure of AlPCS_4_ phthalocyanine and fluorescence auto-correlation curves of free AlPCS_4_ and of its complex with the PAMAM dendrimer G4 (top). The electrostatic potential on the solvent accessible surface of AlPCS_4_ (left side) and of the PAMAM G4 dendrimer (right side) showing all terminal amines protonated and condensed chlorine counterions—red and blue indicates negatively and positively charged regions, respectively. Reproduced from [24] with permission from the PCCP Owner Societies.

Porphyrins have a natural tendency to self-assemble through π-π stacking and other intermolecular interactions. Throughout this process, nanometric structures are generated with interesting optical and electronic properties, distinct from those of the monomer [18,19]. Anionic *meso*-tetrakis (4-sulfonatophenyl)porphyrin (TPPS) interacts with poly(amido amine) (PAMAM) dendrimers of generations 2.0 and 4.0 (G2 and G4, respectively) in an aqueous solution to form several dendrimer-associated species, which depend on the relative concentrations of dendrimer to porphyrin (D/P ≡ [PAMAM]/[TPPS]). At D/P ratios above the isoelectric point of charge balance between the dendrimer and the porphyrin, an equilibrium was observed between TPPS H-aggregates and dendrimer-associated TPPS monomer species that was studied by means of steady-state and time-resolved fluorescence emission. At low pH, the dendrimer provides a molecular template that favors extensive J-aggregation, which induces the appearance of shifted bathochromic bands as regards both Soret and Q-bands, and a pronounced shortening of emission lifetimes. Later, the interaction of porphyrins with PAMAM dendrimers in an aqueous solution was extended to other examples of charged porphyrins, thereby confirming the role of the dendrimer template in the self-association of excitonic aggregates [20]. Aluminum phthalocyanine associates with PAMAM dendrimers of the integer generation in aqueous solutions due to electrostatic and hydrophobic interactions [21]. Single-molecule fluorescence of the association complex between the phthalocyanine and PAMAM dendrimers, as measured in surface-immobilized polymer films, revealed both static and dynamic fluorescence quenching due to electron transfer from the dendrimer tertiary amines to the excited-state phthalocyanine, similarly to a quenching process also found on porphyrin–dendrimer complexes [22,23]. Single-molecule fluorescence measurements gave direct access to heterogeneity in the quenching effect, particularly through the analysis of single-molecule traces that revealed a correlation between intensity and lifetime changes (Figure 1a). Apparent binding constants of PAMAM dendrimer–phthalocyanine complexes in aqueous solutions were later determined by measuring their diffusion times using the Fluorescence Correlation Spectroscopy (FCS) technique (Figure 1b) [24]. 

**Figure 2:**
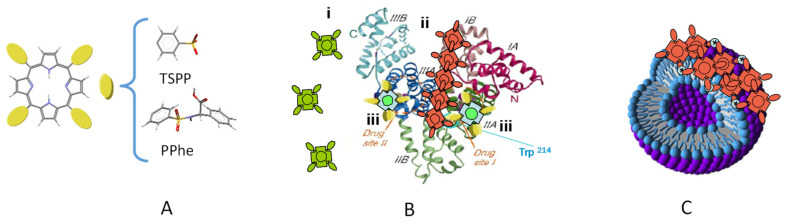
(**A**) Molecular structure of *meso*-substituted porphyrins. TPPS interaction with (**B**) HSA and (**C**) DMPC vesicles: Monomers (green), J-aggregates (red), TPPS–HSA complex (yellow). Adapted with permission from [27] Copyright © 2008, John Wiley and Sons.

A non-covalent complex was reported for the TPPS interaction with albumins (HSA and BSA) [25,26,27] due to the favorable electrostatic interactions between the porphyrin sulfonate groups and the positively charged aminoacid lateral groups (e.g., lysines) of the albumin binding site (in subdomains IIA, Figure 2), confirmed by the observed induced circular dichroism (CD) of TPPS. This complex, prevalent at low porphyrin/protein ratios, was destabilized at higher ratios by lowering the aqueous solution pH (from neutral to acidic) leading to the porphyrin self-assembling in a head-to-tail configuration (J-aggregate) stabilized by the positive charge of the protein surface. Under these conditions, TPPS J-aggregates were also detected using FCS through an increase in molecular brightness [26]. Using the same technique, it was possible to show that TPPS induces conformational changes that stabilized HSA towards denaturation by chaotropic agents or at low pH, highlighting the possibility of TPPS acting as a reporter of HSA conformational changes [28]. In contrast to TPPS interaction with 1,2-dimyristoyl-sn-glycero-3-phosphatidylcholine (DMPC) vesicles, where J-aggregates of the porphyrin were induced at the vesicle surface (Figure 2C), free base tetraphenyl porphyrin with sulfonamide substituents linked to the aromatic amino acid, phenylalanine, in *meso*-positions (PPhe, Figure 2A), self-associated into a co-facial arrangement that was destabilized due to competing hydrophobic interactions with the DMPC bilayer and consequent monomer incorporation [29]. 

Several reports regarding the effects and the role of the solvent medium on the structure and morphology of porphyrin aggregates were reported [30,31,32,33]. 

**Figure 3:**
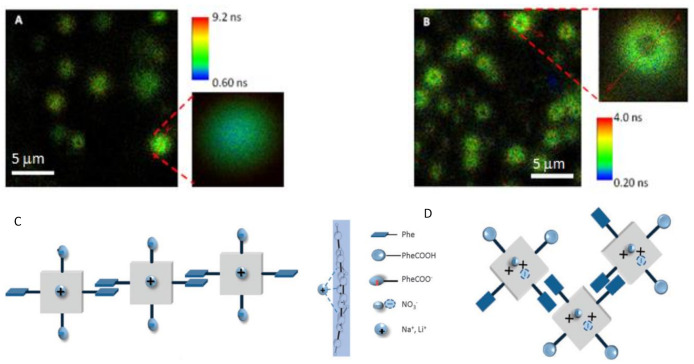
Representative FLIM images and amplifications of marked regions of (**A**) DiCPP-opp at pH = 12 (NaOH) and of (**B**) DiCPP-adj at pH = 0.8 (HNO_3_). Possible models for the aggregates’ structure: (**C**) End-to-end porphyrinic arrays in DiCPP-opp, pH = 12 (front and side views), and (**D**) layer-by-layer porphyrinic arrays in DiCPP-adj, pH = 0.8. Reproduced from [34] with permission from the Centre National de la Recherche Scientifique (CNRS) and The Royal Society of Chemistry.

Functionalization of porphyrins with appropriate peripheral substituents can also result in the formation of porphyrin aggregates with different size and shapes displaying well-defined morphologies. *Meso*-dicarboxyphenyl porphyrins DiCPP-opp and DiCPP-adj form spontaneously pH-induced aggregates in aqueous solutions (Figure 3) [34]. At pH = 0.8, the adjacent carboxylic groups in DiCPP-adj are not charged and the counterion (NO_3_^−^) plays a crucial role in the establishment of highly-ordered J-aggregates mediated either by the nitrate anion or by hydrogen bonding. Conversely, at pH = 12, both the effects of the metal counterion as well as the stacking of opposite phenyl groups in DiCPP-opp favor aggregation. The aggregates were detected by electronic absorption and emission, fluorescence lifetimes, dynamic light scattering, and circular dichroism. The fluorescence lifetime imaging microscopy, FLIM, shows ring aggregates of DiCPP-adj at pH = 0.8 (Figure 3B), which are likely associated with a helicoidal structure. At pH = 12, DiCPP-opp arranges end-to-end resulting in circular-type aggregates detected by the same spectroscopic technique (Figure 3A). The type of molecular architectures and the extent of aggregation are related to the relative positions of the 4-carboxyphenyl units attached to the porphyrin core (Figure 3C,D).

Novel amide-linked porphyrin-rhodamine dyads were synthesized and investigated. Both porphyrin and rhodamine moieties were covalently linked through an amide, which enhances the energy delocalization without prior self-assembly. The charge transfer within the dyads is dominated by the full electronic conjugation shown by intense red-shifted electronic absorption beyond the near-infrared region [35]. 

Protoporphyrin IX and amino acid derivatives were shown to possess amphiphilic properties, which arise from the large hydrophobic surface and from two peripheral chains with ionizing carboxylic groups. The possibility to add alkyl-chains of variable length or amino acid groups or peptides can be used to explore its capabilities to interact with lipids [36,37,38]. The aggregation behavior was investigated in aqueous solutions, as a function of pH and ionic strength using the spectroscopic techniques UV/vis, fluorescence emission spectroscopy, and resonant light scattering (RLS). In the pH range of 0−3, protoporphyrin IX exists as a monomer; a dimer is present at pH > 8, and higher aggregates are formed within the pH range 3−7. Solutions containing protoporphyrin IX from samples at intermediate pH originate aggregates, which were investigated by scanning electron microscopy (SEM) and scanning near-field optical microscopy (SNOM). The analysis of the SNOM images points to the presence of regions in which the aggregation process resulted in a thin film covering the vesicles. An analysis of SEM experiments also shows the layered structure.

#### 2.1.2. Porphyrinoids in Heterogeneous Assemblies

Cationic polyelectrolytes such as poly-L-lysine (PLL) and poly (allylamine hydrochloride) (PAH) also played a crucial role in contributing to the debundle and dispersion of multiwall (MWCNT) and single-wall carbon nanotubes (SWCNT) in aqueous solutions. Moreover, using FLIM, it was possible to confirm the existence of porphyrin arrays associated with the CNT sidewalls wrapped in PLL. In the case of SWCNT, due to its stronger tendency to bundle, H-aggregates resulting from π-π stacking of porphyrin units on neighboring nanotubes were detected upon the hybrid film [39]. 

The relevant role of PLL in the porphyrin–carbon material noncovalent interaction was further explored with photoactive assemblies. A *meso*-tetra aryl porphyrin monosubstituted in one of the phenyl groups with a PLL chain was used to potentiate the porphyrin interaction with oxygenated groups in graphene oxide (GO). The efficient quenching of the porphyrin luminescence in the composite demonstrated the important interactions between the excited state of the porphyrin with GO, which were absent when AlPcS_4_ was used as a fluorophore due to the strong electrostatic interactions with GO for the latter. A different behavior was observed in the presence of gold nanoparticles (AuNP) grown on the GO surface, where a concomitant enhancement of the phthalocyanine fluorescence was detected both in solution and in films, using FLIM. In the porphyrin case, the plasmonic enhancement is less favored due to the stronger interaction with GO that does not allow for the suitable porphyrin-AuNP distance for the metal-enhanced fluorescence (MEF) process [40]. 

#### 2.1.3. Porphyrinoids’ Peptide Conjugates

The precise arrangement of porphyrin-containing peptide conjugate systems is very important for their corresponding role in the biological reactions in nature [41]. Self-assembled nanostructures of a porphyrin−pentapeptide conjugate in water/THF mixtures were investigated as regards their morphology and chirality. The results obtained revealed the effect of the peptide secondary conformation and provided important insights toward the understanding of aggregation behavior of peptide-containing porphyrins occurring in natural systems [42]. The self-assembly of MGG, a neutral *meso*-methoxy phenylporphyrin functionalized with a dipeptide (glycylglycine) substituent (MGG) in water and in water–ethanol mixtures, was investigated by absorption and fluorescence spectroscopy. In water, hydrophobic interactions and the noncovalent intermolecular hydrogen bonding between the terminal carboxylate group of one porphyrin and the hydrogen atoms of the pyrrolic nitrogen atoms of another porphyrin originated non-specific disorganized H- and J-aggregates. The addition of small amounts of ethanol (0.1–25% *v*/*v*) to the water created small “clusters” within which porphyrin J-aggregates reorganize as revealed by a narrow intense band detected by the Rayleigh light scattering (RLS) at 443 nm. The formation of excitonic-coupled supramolecular MGG structures of brickwork or staircase types was proposed in these water–ethanol mixtures [43]. 

The exciting possibility of using reversible bonding among structural units to design functional soft materials is being intensively explored [44]. These materials have the ability to adapt and change in response to their environment or to external stimuli, which, combined with photostability or the integration of many functionalities, makes these materials extremely interesting for multi-purpose applications [17,45]. The construction of biomimetic nanoarchitectures is a particularly promising strategy that exploits the self-assembly of suitably functionalized short amino acid sequences. Assemblies of bioinspired designed peptides can display structural and mechanical robustness and yet can be controllable and reversibly disassembled, which provide for remarkable functional behaviors. In this sense, the self-assembly of peptides is a powerful bottom-up approach for the synthesis of nanomaterials with complex, hierarchal architectures [46,47,48,49]. Pyropheophorbide-*a* (PPh), which is a free base derivative of chlorophyll *a*, was used as the absorbing chromophore with both bands non-overlapping in PPh for symmetry reasons [50] together with the associated vibronic sidebands, giving rise to a rich pattern of absorption bands in the range of 500–700 nm, which can usually only be attained by mixing more than one chromophore [51]. This is a particularly desired feature toward the realization of ideal panchromatic light harvesters.

The tracking and monitoring of fundamental biological processes without complicated and potentially toxic labeling can be made with bioorganic molecules with intrinsic fluorescence [52,53,54,55,56,57]. As a natural ingredient of biological systems, peptides are strong candidates for self-assembly through extensive and directed hydrogen bonding, and aromatic interactions, thus prompting extensive efforts to channel these features into the development of the next-generation of functional biomaterials [58,59,60,61,62,63,64,65,66,67,68,69,70]. One noticeable example is diphenylalanine that self-assembles into diverse nanostructures potentially useful in the biomedical field for biosensing. Initially, diphenylalanine was identified as the smallest core recognition the motif of β-amyloid, the amyloidogenic polypeptide associated with Alzheimer’s disease [71,72,73]. However, the potential application of intrinsically fluorescent peptides as eco-friendly materials for optoelectronic devices is severely limited by their low quantum yields and low photostability, which has prompted their derivatization with porphyrinoid molecules [74]. 

Green fluorescent protein (GFP) has been extensively used as a genetically encoded fluorescent marker in biology [75]. Specifically, a GFP mutant (BFPms1) that preferentially binds to Zn(II) was developed to seek enhancements of its emission. The binding of Zn(II) rigidifies the chromophore imidazole, thus reducing its mobility, and inhibiting energy dissipation through thermal relaxation pathways, which enhances fluorescence emission from this GFP mutant [76]. Inspired by this molecular structure, a metal-binding site linked to a short peptide can simulate metal coordination. An electrostatic interaction site might generate supramolecular hosts of a β-fold barrel environment for self-locking. In this way, it was possible to fabricate a minimalist version of this sophisticated biological structure, which will provide a scalable technological solution. The coordination of metal ions in enzymes can be mimicked by cyclic peptides, which are derived from amino acid residues carrying complex side chain substituents, such as imidazole, carboxylate, or thioether groups [77,78,79,80,81,82]. Furthermore, cyclic dipeptides are versatile molecular motifs due to the hydrogen bonding capabilities of the skeleton and other noncovalent interactions that can be used to design multifunctional scaffolds [83,84]. 

The assembly of cyclic(l-histidine-d-histidine) (CHH) was explored to construct highly fluorescent peptide dots with a large quantum yield (>0.7) through the “self-assembly locking” strategy [77]. In this work, it was demonstrated that CHH self-assemblies showing a bright fluorescence, which may be integrated as an emissive layer in the photo- and electroluminescent light-emitting diodes (LEDs). The “self-encapsulation” strategy was followed to construct a nanocarrier delivery system to transport an anticancer drug into cancer cells while monitoring the delivery process in situ.

Molecular self-assembly on surfaces enables the surface structure with particular and specific functionalities [85]. Adjusting the balance between attractive and repulsive forces, it is possible to devise a strategy to extend the structural variety. Important examples of MSA in materials include the formation of molecular crystals, colloids, lipid bilayers, and self-assembled monolayers (SAM) [86]. These systems constitute the self-assembly of organic π-conjugated molecules into nanostructured assemblies, which are attracting attention towards the development of functional materials for electronic and biological applications just using an easy potential and effective route. Recently, new supramolecular structures, finite-size networks, and porphyrin-decorated brushes were obtained [87,88]. 

### 2.2. Microencapsulation

**Figure 4:**
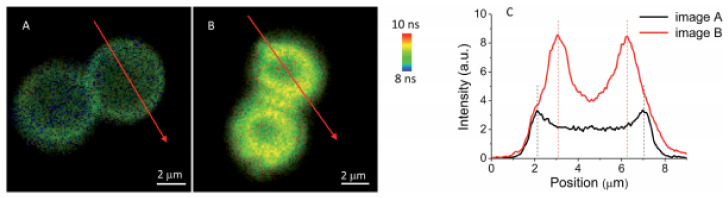
Microcapsules with the sequence (PAH/PSS)_5_-(PAH-TCPP)-PSS in water at: (**A**) pH = 5.6; and (**B**) pH = 7.0. (**C**) Intensity profiles of the red arrows in images (**A**) and (**B**). Reproduced from [89] with permission from The Royal Society of Chemistry.

During the last decades, the development of micro- and nano-systems attracted enormous attention for applications in such different areas from sensor devices to target gene therapy. The possibility of using such structures as carriers for cargo delivery opened up the opportunity for applications in biomedicine and encouraged the development of new biocompatible devices and materials. A promising alternative to those systems was the polymeric micro/nano capsules because they offer many possibilities that potentially meet the criteria of stability, biocompatibility, high loading capacity, and versatility in the fabrication of the capsule shell. In particular, the surface and the inner layers can be easily functionalized with organic or inorganic compounds tuning their inner composition so that they can perform different functions. It is envisaged that a wide diversity of chemical species, such as proteins, nucleic acids, inorganic nanoparticles, or dyes can be incorporated into the shell structure creating materials with unique tailored properties, which make these systems very attractive for a wide range of applications. The most common pair of charged polyelectrolytes (PEs) are the cationic PAH and the anionic PSS, poly(styrene sulfonate). The known ability of porphyrins to induce the formation of singlet oxygen and related oxygen species under light exposure leads to the destruction of the capsule walls and the release of the active component. The incorporation of porphyrin and phthalocyanine dyes with photo-, thermo-, or electrochemical properties in the capsule shells can have potential interest for the preparation of light harvesting, remote release systems, and microspheres laser approaches. Microcapsules carrying porphyrins and phthalocyanines can also be interesting micro- or nano-reactors to perform, for instance, photocatalytic reactions. In that case, higher amounts of incorporated fluorophores could be obtained by the adsorption of molecules to several layers, which would likely improve the efficiency of photocatalysis. Porphyrin encapsulation in these systems was demonstrated by fluorescence lifetime imaging microscopy (FLIM, Figure 4), steady-state fluorescence, and time-resolved fluorescence single-photon counting [89]. 

**Figure 5:**
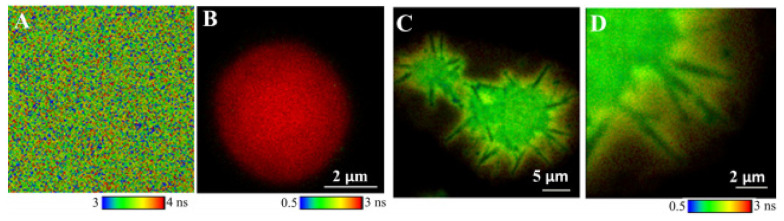
FLIM images of (**A**) TPPS in water (pH 3.0), (**B**) hollow (PAH/PSS)_2_PAH, and (**C**) core-shell CaCO_3_ (PAH/PSS)_2_PAH PECs after TPPS adsorption (pH 3.0). (**D**) Magnification of C. λ_exc_ = 483 nm. Adapted with permission from [90]. Copyright 2017 American Chemical Society.

More detailed information can be assessed by FLIM, which emerged as a key technique to discriminate different fractions of the same fluorophore in different states of interaction with the environment. The molecular effects of the unknown and usually variable fluorophore concentration can thus be independently investigated. An inorganic core-assisted strategy was developed to promote the organized self-assembly of TPPS on pH-sensitive polyelectrolyte microcapsules (PECs) made of PAH and PSS polyelectrolytes [90]. A key feature of this strategy was the use of template CaCO_3_ micro-particles as a nucleation site endorsing inside–outside directional growth of porphyrin aggregates. With this approach, TPPS self-assembled in positively charged CaCO_3_ (PAH/PSS)_4_PAH. PECs were successfully achieved using mild pH conditions (pH 3). Evidence for porphyrin aggregation was obtained by UV-Vis due to the presence of its characteristic absorption bands in PECs functionalized with porphyrins at pH 3. FLIM of the polyelectrolyte core shell confirmed the presence of radially distributed needle-like structures sticking out of the polyelectrolyte shells associated with short fluorescence lifetimes (Figure 5C,D), indicating a strong electronic coupling between porphyrin units. Microscopic images also revealed a sequential process for the directional growth (inside/outside) of TPPS aggregates (adsorption, re-distribution, and aggregation) and highlight the importance of the core in aggregation induction. Removing the CaCO_3_ core alters the porphyrin interaction in the PECs environment, and aggregate growth is no longer favored (Figure 5B). Considering these features, the application of these systems in the development of light-harvesting systems may be interesting.

The conjugation of FLIM and FCS techniques showed that the aggregation/de-aggregation pattern of AlPcS_4_ and of chlorophyll *a* microencapsulated in spontaneous colloidal structures of DMPC and DTAC (i.e., dodecyltrimethylammonium chloride) was greatly affected by the colloidal architecture media. This was dependent on the detergent/lipid ratios (D:L), which rendered multi- and unilamellar vesicles at D:L ≈ 2 and formed disks, bilayers, or thread-like micelles at D:L ≈ 15–20. While J- and H-aggregates prevailed in these structures, only monomers were detected in DTAC micelles [91,92]. 

### 2.3. Imaging Microscopy

The photoluminescence of porphyrins and related tetrapyrrolic compounds has been vastly explored for imaging microscopy, in particular, in the development of multifunctional platforms for applications in the fields of biomedical imaging or photomedicine [93,94,95,96,97,98]. Therefore, many of the selected examples herein presented concern imaging microscopy of supramolecular or nanostructured assemblies of porphyrin-like compounds that have been proposed in the literature for biomedical applications such as PDT, photothermal therapy (PTT), drug delivery, bioimaging, sensing, among others. The role of porphyrin-based systems in other medical non-optical imaging modalities, such as positron emission tomography (PET) or magnetic resonance (MR) imaging, despite being widely recognized, does not fall into the scope of this section, and has been reviewed in the literature [99]. Other examples of porphyrin’s emission in optical imaging have been reported from research fields, such as natural or artificial photosynthesis, photocatalysis, and optical sensors. Although here the literature volume is more modest, a few selected examples will be presented.

**Figure 6:**
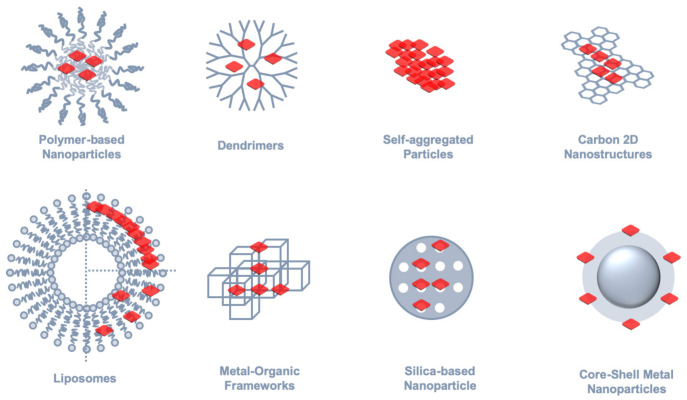
Examples of supramolecular assemblies and nanostructures involving porphyrins, phthalocyanines, and their analogues that have found application in imaging microscopy.

The inclusion of porphyrins or related tetrapyrrolic macrocycles into supramolecular or nanostructured assemblies is generally sought as a strategy for expanding the already-remarkable properties of these compounds. In terms of optical properties, the controlled assembly of porphyrins opens the way to explore excitonic coupling for tuning the absorption spectrum, e.g., to achieve shifted bathochromic Q-bands, or it may serve to improve emission intensity through aggregation-induced emission enhancement (AIE). For some specific applications, such as PDT or drug delivery, the assembly into supramolecular entities is a valuable approach for overcoming solubility issues or to improve pharmacokinetic properties by building in active targeting or controlled release strategies. Examples of supramolecular architectures used for porphyrin inclusion are depicted in Figure 6 and selected works covering some of these examples will be discussed next in the context of imaging microscopy.

#### 2.3.1. Polymer-Based Nanoparticles

**Figure 7:**
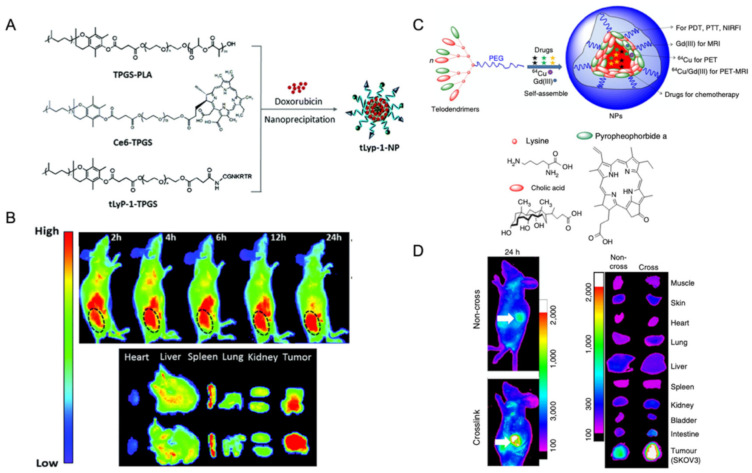
(**A**) Polymer-based nanoparticles from Ce6-labeled D-α-tocopheryl polyethylene glycol 1000 succinate (Ce6-Table 7). (**B**) ADR cells intravenously treated with Ce6-labeled tLyp-1-NPs through the tail vein at 2 h, 4 h, 6 h, 12 h, and 24 h, respectively, showing that Ce6-labeled Lyp-1-NPs were detected in the organs and tumors 24 h post-administration. Reproduced from [104] with permission from The Royal Society of Chemistry. (**C**) Polymer nanoparticles self-assembled from a porphyrin–telodendrimer composed of four pyropheophorbide-a molecules and four cholic acids attached to the terminal end of a linear PEG chain. (**D**) In vivo NIR fluorescence imaging of nude mice bearing SKOV3 ovarian cancer xenograft following intravenous injection of polymer nanoparticles non-crosslinked and disulpide-crosslinked. The white arrow points to the tumor site. Representative ex vivo NIR fluorescence imaging of SKOV3 ovarian cancer xenograft 24 h post injection of non-crosslinked (left) and disulpide-crosslinked (right) polymer nanoparticles [103]. Copyright © 2014, Nature Publishing Group, a division of Macmillan Publishers Limited. All Rights Reserved.

Polymer-based nanoparticles that are assembled from amphiphilic polymers form micellar entities that can reach sizes from tens to hundred nanometers. The incorporation of tetrapyrrolic macrocycles into these nanoparticles has been achieved both in a covalent or non-covalent fashion [100,101,102,103,104]. This approach has been used to develop multifunctional platforms for nanomedicine, usually combining porphyrin-sensitized PDT with chemotherapy for tumor treatment and, furthermore, taking advantage of porphyrin’s emission for in vivo imaging of drug agents to monitor their dissemination in the organism of animal models. The typical emission of porphyrins and their analogues in the near infrared (NIR) range of the electromagnetic spectrum is particularly advantageous, because it falls in the biological window, thus providing deeper penetration of light into biological tissues for PDT/PTT and bioimaging purposes. In Figure 7A, an example of a polymer-based nanoparticle is highlighted in which a porphyrin analogue, chlorin e6 (Ce6), was covalently linked to D-α-tocopheryl polyethylene glycol 1000 succinate (Ce6-TPGS) and formulated into micelle-like assemblies by mixing with a poly(lactic acid) derivative (TPGS-PLA) [104]. By means of in vivo near-infrared imaging of mice bearing subcutaneous MCF-7/ADR cells—a multidrug-resistant breast cancer cell model—it was possible to show that polymer nanoparticles labelled with Ce6 are preferentially accumulated in tumorous tissues when decorated with tLyp-1 peptide for active targeting (Figure 7B). In another example, polymer nanoparticles were assembled from telodendrimers bearing another porphyrin analogue, Pyropheophorbide-*a*, which may perform multiple functions of PDT/PTT and NIR imaging (Figure 7C) [103]. The hydrophobic core of the micelle assemblies can also be loaded with other agents for non-optical imaging, such as PET or MRI. In this work, the NIR emission of Pyropheophorbide-*a* was used to monitor its biodistribution upon intravenous injection of polymer nanoparticles in nude mice bearing SKOV3 ovarian cancer xenograft. It was possible to verify a selective accumulation effect at the tumor site via the size-mediated enhanced permeability retention (EPR) effect. Furthermore, it was shown that disulphide-crosslinked nanoparticles had a prolonged and more localized release of the porphyrinoid PDT agent, as promoted by the endogenous reducing agent glutathione (GSH) at the tumor site (Figure 7D).

#### 2.3.2. Dendrimers

Dendrimer nanocarriers are a particular type of polymer-based nanoparticles, in which a highly branched and regular polymer architecture may be conceptualized as a unimolecular micelle [105]. Dendrimer scaffolds are often used either as a template for the supramolecular assembly of organic dyes, or as a nano-container for their encapsulation [18,19,106,107,108,109,110,111,112,113,114]. The latter approach has been employed using phthalocyanines, which are an artificial analogue of porphyrins, that perform both as photosensitizers for PDT and as dye labels for cellular and in vivo imaging. In this specific example [111], a silicon phthalocyanine was modified with a hydrophobic linker to promote its loading into the core of a generation 4 poly(propylene imine) (PPI G4) dendrimer. The dendrimer was further modified with poly(ethylene glycol) (PEG) and a luteinizing hormone-releasing hormone (LHRH) peptide, in order to improve biocompatibility and tumor-targeted delivery. The NIR emission of encapsulated phthalocyanines was used to track subcellular localization in vitro and organ distribution in vivo using mice bearing ovarian cancer xenograft as an animal model. Later, the same group incremented this work by replacing the dye with a naphthalocyanine, which has an extended π-system and, thus, emits farther in the NIR range [112]. 

The molecular aggregation of porphyrin-like compounds into nanometer-sized particles that have a core exclusively composed of dye molecules is another approach that explores the high planarity of the tetrapyrrolic macrocycle to enable extensive π-π stacking [115,116,117,118]. The paradigmatic case of TPPS that, in the diacid form, self-organizes into J-aggregates with well-defined spectroscopic signatures of excitonic coupling has been extensively reported and employed in the design of supramolecular architectures [18,19,25,28,29]. In general, excitonic interactions between aggregated dyes allow, to a certain degree, tuning the spectral and photodynamic properties of these aggregates, which would only be fully accomplished by precise control over distance and orientation between monomer units [119]. On the other hand, the phenomenon of AIE may also affect the photodynamics of aggregated dyes either through suppression of low-frequency modes involved in non-radiative decay channels—that may also involve a shielding effect from solvent—and/or planarization of the π-system of aggregated dyes [120]. The development of porphyrin nanoparticles displaying AIE has been shown to provide improved biological imaging capabilities. However, the peripheral substituent groups of the porphyrin core play a crucial role [116], as a zinc tetra methylphenyl porphyrin showed aggregation-induced quenching, while a porphyrin decorated with tetraphenyl ethene peripheral groups resulted in the desired AIE effect.

#### 2.3.3. Carbon 2D Nanostructures

The integration of tetrapyrrolic macrocyles with electron-rich 2D carbon nanostructures has been pursued to develop nanocomposite materials with exceptional charge transport properties that can be optically addressed in the visible spectral range [39,40,121,122,123]. Although to a lesser extent, the application of these nanocomposites for bioimaging or chemical sensing has also been explored [124,125,126]. For instance, the photosensitizer Ce6 has been loaded onto PEG-functionalized GO via supramolecular π-π stacking to develop a PDT agent, that was demonstrated to have a significantly enhanced intracellular trafficking, when compared to free Ce6, thus offering improved cancer cell photodynamic destruction [124]. Besides, it was also shown that the graphene-mediated photothermal effect provided for a controlled release of Ce6 by using a near-infrared laser at a low power density, thus increasing the efficacy of PDT treatment.

#### 2.3.4. Liposome Nano-Assemblies

Lipid-based supramolecular structures, and among these, liposomes—that are nanometer-sized vesicular assemblies—have been successfully employed in clinical practice as vehicles for drug delivery [127,128]. A contemporary example in the context of the COVID-19 pandemic is that of mRNA vaccines that were among the firstly approved for general public use and that have a liposome-base formulation [129]. The encapsulation of porphyrins and related tetrapyrroles into liposome nanostructures seeks to explore their role as photosensitizers in PDT for cancer treatment [130], age-related macular degeneration [131], dermatologic diseases [132], and microbial infections [133]. Typically, the nonpolar region within the lipid can solubilize hydrophobic porphyrins, then employed for biomedical applications. 

**Figure 8:**
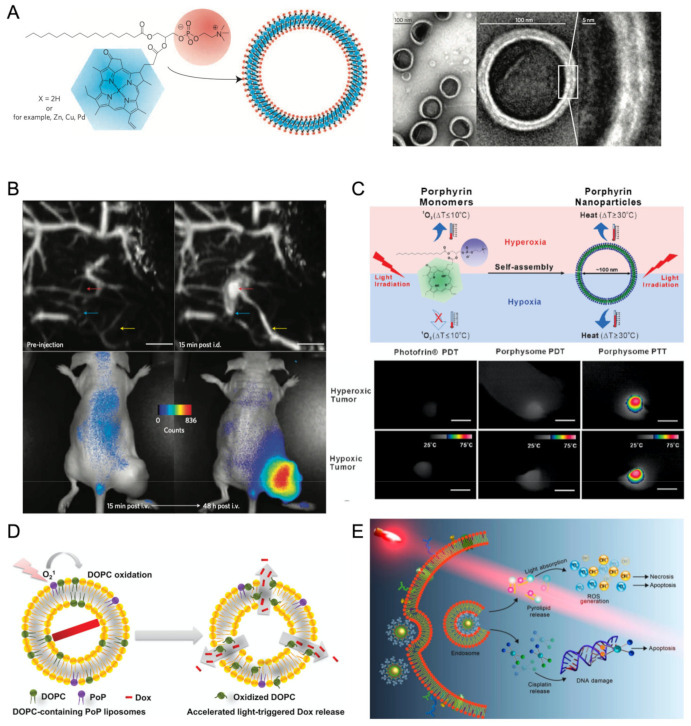
(**A**) Schematic representation of a pyropheophorbide–lipid porphysome highlighting the phospholipid headgroup (red) and porphyrin (blue) in the PoP subunit and assembled nanovesicle (left), and electron micrographs of negatively stained porphysomes (right) [136]. Copyright © 2011, Nature Publishing Group. (**B**) Dual modality for photoacoustic contrast and activated fluorescence: Lymphatic mapping in rats using photoacoustic tomography before and after intradermal injection of porphysomes (top left and right panels, respectively); fluorescence activation after intravenous injection of porphysomes in a KB xenograft-bearing mouse for 15 min and 48h time delays (bottom left and right panels, respectively). Adapted with permission from [136]. Copyright © 2011, Nature Publishing Group. (**C**) Scheme of nanostructure-driven conversion from the PDT singlet oxygen generating mechanism of porphyrin to a completely thermal mechanism based on fluorescence self-quenching, which can be employed for PTT enhancement (top). Temperature increase upon laser irradiation captured by a thermal camera in hyperoxic and hypoxic tumors subjected to various irradiations with: Photofrin PDT, porphysome PDT, and porphysome PTT (scale bar: 8 mm) [137]. Copyright 2013 American Chemical Society. (**D**) Scheme of light-triggered Dox releasing from PoP liposomes, which is induced by DOPC oxidation due to singlet oxygen generation upon irradiation [138]. © 2016 WILEY-VCH Verlag GmbH & Co. KGaA, Weinheim. (**E**) Scheme showing endocytosis of NCP@pyrolipid and subsequent apoptosis/necrosis by combined chemotherapy and PDT. Adapted with permission from [139]. Copyright 2015 American Chemical Society.

A new paradigm was introduced by Zheng and co-workers that, by covalently linking porphyrins onto phospholipids, conceived a specific porphyrin–phospholipid (PoP) building block for liposome assemblies that were coined as porphysomes (Figure 8A) [134,135]. One important criterion in the design of PoP blocks was that their components and metabolic products were already clinically studied macromolecules or were demonstrated to be safe in pre-clinical studies. Moreover, the porphyrin’s emission provides a molecular imaging functionality that can be explored as an investigational tool in the early stages of therapy development. Porphysome platforms may contain exceptionally high concentrations of a photosensitizer, as it was estimated that a single spherical 100-nm pyro-lipid porphysome may load approximately 80,000 light-active porphyrins subunits. The high local concentration paves the way for a strong self-quenching effect that, despite being detrimental for fluorescence imaging, was employed for photoacoustic tomography (Figure 8B) and for PTT treatment in animal models (Figure 8C) [136,137]. The formulation of porphysomes with a minor fraction of folate–PEG–lipid, in order to promote uptake by cancer cells using a folate-receptor-targeted strategy, showed that upon cell internalization, fluorescence emission is triggered by the dissociation of PoP subunits, which allowed for multimodal imaging using a single nanoplatform for both photoacoustic tomography and low-background fluorescence imaging. 

The porphysome architecture also intrinsically provides for a mechanism of light-triggered drug release, in which singlet oxygen generation by PoP subunits has been proposed to drive an oxidation-induced process of the liposome bilayer that upon disruption releases cargo molecules loaded into the aqueous inner compartment (Figure 8D) [138]. In vitro assays have demonstrated that the loaded drug can be released in a minute under NIR irradiation, while in vivo assays using a human pancreatic xenograft in mice have shown a significant growth delay upon light treatment with porphysomes carrying the doxorubicin (Dox) chemotherapy drug, when compared to treatment with the same dose of Dox–loaded porphysomes without irradiation, or empty porphysomes with light treatment. In another example, the porphysome inner pool has been exploited for loading a nanoscale coordination polymer (NCP), thus resulting in a core-shell architecture, in which the NCP carries high payloads of cisplatin and the liposome bilayer carries PoP photosensitizer subunits for combined chemo- and phototherapy (Figure 8E) [139]. 

Other core-shell architectures of porphysomes have been designed to incorporate up-conversion nanoparticles (UCNPs) at the core compartment, which further expanded the emission signals available for imaging microscopy in a multimodal approach that also included non-optical imaging modalities [140]. Other formulations of core-shell nano-assemblies comprising porphyrin-like compounds and UCNPs for combined PDT and imaging microscopy functionalities have been reported in the literature [141,142,143]. The role of porphyrins in nonlinear optical imaging will not be further covered here, but the interested reader is referred to the dedicated literature [144]. 

#### 2.3.5. Metal Organic Frameworks and Silica Nanoparticles

Hybrid nanomaterials that combine organic with inorganic components have been extensively used to incorporate porphyrin units envisaging their application in imaging microscopy. Metal organic frameworks (MOFs) are a prime example of such nanomaterials, in which the ability of tetrapyrroles to coordinate metal ions in the macrocyle’s central position provides an intrinsic structural motif for building regular 3D structures [145]. It was shown that nanoscale porphyrin MOFs (NPMOFs) can be prepared from biocompatible zirconium ions and *meso*-tetrakis(4-carboxyl)-21H,23H-porphine using the microemulsion method to control the particle size. The pore structure of NPMOFs was further employed to load Dox molecules for a dual chemo- and phototherapy approach. The red fluorescence emission from porphyrin units was preserved in the NPMOFs, which provided for a means of fluorescence image-guided therapy taking advantage of the high penetration depth in the biological window, as validated in cancer cells, zebrafish, and mice models, used therein for cancer imaging and drug tracking [146]. 

Another class of organic-inorganic hybrid nanomaterials that have been conjugated with porphyrins and their analogues are silica-based nanoparticles. In this example [147], the synthesis of biocompatible and hydrophilic silica–porphyrin hybrid nanotubes (HNTs) was achieved by a sol–gel method using a cationic organoalkoxysilane and an anionic porphyrin, tetrakis (4-carboxyphenyl)porphyrin (TCPP). These components were self-assembled via π–π stacking and electrostatic interactions, which resulted in the formation of porphyrin J-aggregates that are characterized by red-shifted absorption and emission bands. The near-infrared emission from porphyrin J-aggregates enabled to use HNTs as fluorescent probes for in vivo imaging, thus providing for high-sensitive detection of these probes in a mouse animal model. 

#### 2.3.6. Core-Shell Metal Nanostructures

**Figure 9:**
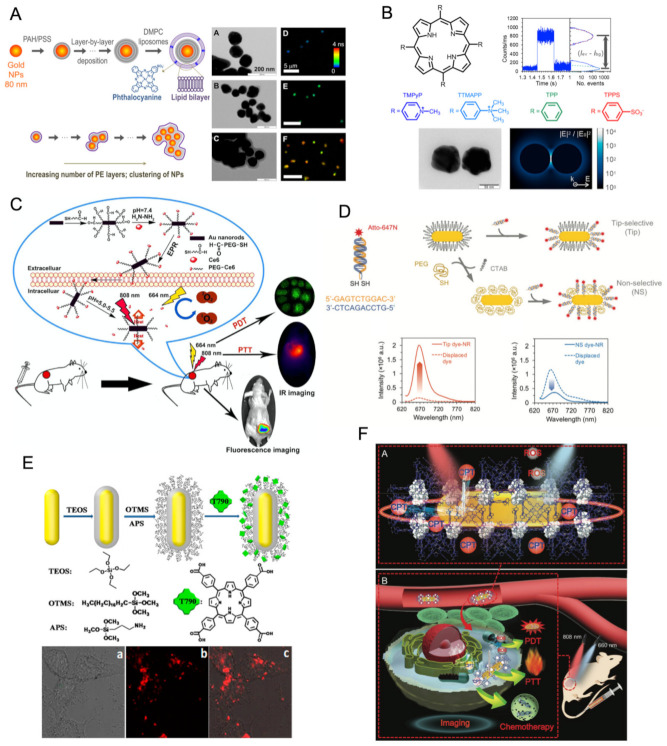
(**A**) Sequence of core-shell preparation of gold nanoparticles with a polyelectrolyte shell of PAH/PSS layers and an outer DMPC lipid bilayer embedding a phthalocyanine dye (top left). Gold nanoparticles form clusters as the number of polyelectrolyte layers is increased (bottom left). Electron microscopy (TEM) and fluorescence lifetime (FLIM) images of gold nanoparticles modified with a polyelectrolyte shell for an increasing number of PAH/PSS layers (5, 9, and 21 layers from the top down). Adapted with permission from [155]. Copyright 2015 American Chemical Society. (**B**) Chemical structure of the four porphyrins investigated for plasmon-enhanced fluorescence. Intensity time trace (top left) and example of an event of extreme fluorescence enhancement for TMPyP porphyrin (top right). TEM images of a dimer of gold nanoparticles obtained by DNA-directed assembly and near-field enhancement map calculated from discrete dipole approximation simulations. Adapted with permission from [156]. Copyright 2019 American Chemical Society. (**C**) Synthesis of core-shell hybrid gold nanorods Ce6-PEG-AuNR and their function for combined PTT/PDT [157]. © 2017 Elsevier B.V. All rights reserved. (**D**) Scheme of dye-labelled dsDNA oligonucleotide and preparation of dye-particle nano-assemblies using: (top) tip-selective thiol attachment toward the nanorod tips; (bottom) non-selective coating process with thiolated DNA oligonucleotides. Emission spectra of tip-functionalized (red) and non-selective (blue) dye-particle nano-assemblies and of dye-labelled oligonucleotides displaced into solution (dashed lines) showing an effective emission enhancement for tip-selective functionalization. Reproduced from [152] with permission from The Royal Society of Chemistry. (**E**) Preparation of gold nanorod-photosensitizer conjugates with an inner shell of silica that acts as a spacer to avoid metal quenching of porphyrin’s emission, and an outer layer composed of aliphatic chains used to load a porphyrin, T790 [164]. Copyright © 2014, American Chemical Society. (**F**) Scheme of gold nanorods coated with a porphyrinic MOF of Zr_6_(TCPP)_1.5_ that was further loaded with a model drug, camptothecin, as a multifunctional theranostic system for combined PDT/PTT and chemotherapy of tumor [159]. © 2017 WILEY-VCH Verlag GmbH & Co. KGaA, Weinheim.

Hybrid organic–inorganic nanostructures built from a metal nanoparticle core coated with an outer shell that encapsulates porphyrin units is a design strategy that makes use of the remarkable optical properties of metal nanoparticles to develop enhanced nanomaterials for imaging microscopy [93]. The strong interaction of metal nanoparticles with light is derived from localized surface plasmons, which are collective and coherent oscillations of free electrons in the metal that are resonantly excited by the electric field of light and are confined by the particle’s surface [148]. This phenomenon induces large electric fields at optical frequencies in regions close to the particle’s surface, where excitation rates of dye molecules may be accelerated many times—plasmon hot-spots [149,150]. The photophysical rates of a dye in the vicinity of metal surfaces are also affected by the contribution of plasmonic modes to the local density of states that affects both radiative and nonradiative decay rates, in the latter case through resonant energy transfer. Plasmonic manipulation of excitation and decay rates in optimal conditions is able to effectively increase emission photon rates from dye molecules by orders of magnitude [151]. This optical antenna effect of metal nanoparticles has been vastly explored to produce exceptionally strong emitting fluorescent nano-assemblies [152,153]. For instance, we have shown that large fluorescence enhancements can be achieved for a phthalocyanine dye encapsulated on a lipid bilayer around clusters of gold nanospheres coated with a layer-by-layer polyelectrolyte shell (Figure 9A) [154,155]. More recently, we have shown that dimer nanogap antennas obtained from the DNA-directed self-assembly of gold nanospheres afforded emission enhancements up to a one-million-fold increase, thus enabling single-molecule detection of porphyrin’s fluorescence (Figure 9B) [156]. 

Hybrid core-shell metal nanostructures containing optically active porphyrin units have been designed and investigated as multifunctional probes that can deliver a photothermal effect and photoluminescence or singlet oxygen generation for combined imaging and PDT/PTT treatments with prospective applications in the biomedical field. The modification of the metal nanoparticle core with a surrounding shell has been achieved through a diversity of surface chemistry approaches such as chemical attachment of thiol derivatives [157], non-covalent supramolecular assembly [158,159,160], entrapment in a polymer or silica shell [161,162], or even a combination of the previous strategies [163,164]. 

A gold nanorod-photosensitizer conjugate was obtained by labelling Ce6 onto thiolate-PEG chains, then used to covalently modify the surface of gold nanorods (Ce6-PEG-AuNR) [157]. This nano-therapeutic agent was designed to simultaneously deliver PTT/PDT treatments (Figure 9C). In this case, the gold nanorods act as a quencher on the fluorescence of Ce6, instead of enhancing the dye’s emission through an optical antenna effect. Nevertheless, in the intracellular acidic conditions of tumor tissue, the hydrazone bond between Ce6 and PEG is cleaved, which provides for the recovery of specific fluorescence from Ce6 and allows for an eventual smart strategy for precise location of tumors and further cancer cell destruction by combined PTT/PDT. The quenching effect found in this work for Ce6-PEG-AuNR nano-conjugates illustrates that is not enough to simply attach the dye molecules onto the particle’s surface for the metal nanoparticle to perform as an optical antenna and induce emission enhancements on the dye’s emission. We have shown that a tip-specific functionalization in the dye attachment onto gold nanorods, in order to target the plasmon hot-spots at the rods’ tips, was an effective strategy for achieving fluorescence enhancement in the emission from our dye-particle nano-assemblies (Figure 9D) [152]. 

Another important feature in the design of core-shell metal nanostructures for enhanced-fluorescence emission is the distance between the dye and metal surface to impose a separation distance between these two components, which prevents quenching by the metal particle. For this purpose, a silica shell may be grown around the latter as a spacing layer. This approach was used to develop gold nanorod-photosensitizer conjugates that have an inner shell of silica and an outer layer composed of aliphatic chains used to load a porphyrin, T790 (Figure 9E) [164]. These conjugates were used as nano-agents for two-photon cell imaging and for two-photon photodynamic therapy with enhanced efficiency using HepG2 cancer cells as in vitro model. Another illustration of the crucial role of dye-particle distance on plasmon-enhanced fluorescence was provided by our work on gold nanospheres incorporated into a core-polyelectrolyte-shell type of assembly with a coating lipid vesicle encapsulating a phthalocyanine dye [155]. The supramolecular construct with polyelectrolyte layers as spacers between the particle’s surface and the phthalocyanine was designed to control the emission enhancement through the plasmonic antenna effect of gold nanoparticles. Large emission enhancements, of about 3 orders of magnitude, for an optimum number of 13 to 15 polyelectrolyte layers, were attributed to hot-spots formed by the clustering of gold nanoparticles during the process of layer-by-layer deposition of polyelectrolytes.

Porphyrin-based MOFs have also been explored to build core-shell metal nanostructures [159,160]. For instance, gold nanorods have been coated with a porphyrinic MOF of Zr_6_(TCPP)_1.5_ that was further used to load the model drug, camptothecin. This nanoplatform was designed for a combined approach of photodynamic, photothermal, and chemotherapy in cancer treatment [159]. The photothermal effect from the gold nanorod also provided for NIR light-triggered control of a camptothecin release, while the increase in reactive oxygen species, when compared to the porphyrinic MOF alone, was attributed to plasmon mediated hot-electron reduction of water. Porphyrin’s emission from gold nanorod@MOFs assemblies allowed for fluorescence imaging to be used for evaluating their in vivo biodistribution (Figure 9F).

#### 2.3.7. Methods and Applications in Imaging Microscopy

##### Fluorescence Lifetime Imaging Microscopy (FLIM)

The information provided by imaging microscopy can be augmented by a multiparametric approach that includes other experimental observables beyond intensity, such as emission lifetime, polarization, or spectral distribution [165]. In particular, the emission lifetime from a dye label is an absolute measurement, in the sense that, contrarily to emission intensity, it does not depend on excitation intensity, optical setup configuration and detector efficiency, or other instrumental details [166]. Moreover, the emission lifetime of specific dye labels is intrinsically sensitive to environmental variables, such as pH, polarity, or viscosity, and thus these properties can be probed by lifetime measurements [167]. In other cases, the emission lifetime is an observable associated to a molecular ruler interaction—such as resonant energy transfer (FRET), photoinduced electron- or proton-transfer, plasmon-coupled emission—that may be integrated in the studied system through a donor–acceptor entity that probes a structural or dynamic property of that particular system. 

FLIM has been a privileged technique in our group to further investigate the interaction of porphyrinoids in supramolecular and nanostructured systems that include micelles [89,115], liposomes [92], molecular self-aggregates [34,43], Langmuir-Blodgett films [168], dendrimers [21], polyelectrolyte microcapsules [89,90,169], carbon nanotubes [39], graphene oxide [40], and gold nanostructures [150,152,154,155,156], as highlighted in some examples previously described in this review.

The detection of single-molecule fluorescence from porphyrin emitters is challenging because of the quasi-forbidden character of $S_{1}\leftarrow S_{0}$ transition, which results in typically low absorption cross-sections for Q-bands and in modest values of fluorescence quantum yield. This feature has motivated us to investigate the fluorescence enhancement of porphyrin emitters using gold nanodimer antennas [156]. The emission enhancement resulted in unprecedented factors by 10^5^ up to 10^6^-fold in emission increase for the maximum detected photon rates. FLIM was used to discriminate porphyrin’s enhanced emission from the diffraction-limited spots of gold nanodimers by using a time-gated image analysis that selectively plots emission events associated with short decay times, as radiative and nonradiative decay rates of the porphyrin emitter are accelerated by many orders of magnitude due to plasmon-coupled emission.

A typical FLIM implementation is based on a confocal fluorescence microscope operating in time-correlated single-photon counting (TCSPC), while wide-field FLIM is based on frequency domain lifetime measurements or time-gated camera detection [166]. Besides FLIM measurements, confocal fluorescence lifetime microscopy also provides FCS measurements [170]. This powerful technique is particularly suited for studying supramolecular interactions in a fluid environment, in which case imaging would be smeared out by Brownian diffusion. We have employed FCS to evaluate the role of electrostatic interaction in the binding of PAMAM dendrimers and charged phthalocyanines in an aqueous environment [24]. The strong near-infrared emission from phthalocyanines provides for single-molecule sensitivity FCS, contrarily to assemblies of porphyrin-dendrimer because of weak fluorescence emission, which is further aggravated by photoinduced electron-transfer involving the terminal primary amines of PAMAM dendrimers.

##### Optical Sensors for Imaging Microscopy

Supramolecular systems involving optically active porphyrins have also found application as optical sensors in imaging microscopy for the detection of a diversity of chemical targets or environmental variables. For instance, the long-lived phosphorescence of Pt-coordinated porphyrins is sensitive to quenching by environmental O_2_, which has been explored to develop sensor surfaces that detect O_2_ using porphyrin’s emission intensity or lifetime as readout signals [171,172]. In another example, a porphyrin-based hydrogel was employed to monitor subcutaneous oxygenation by in vivo NIR imaging [173]. The phosphorescence emission from a palladium coordinated porphyrin (Pd-mTCPP) is partially quenched by energy transfer to environmental oxygen, which was used to probe transdermal luminescence imaging of dual-implanted Pd-mTCPP and 15% free base-Cu-mTCPP hydrogels, thus showing a ratiometric approach to monitor oxygen levels in a mice model. Although in this work, the use of phosphorescence lifetime in vivo measurements was dismissed as technically challenging, this alternative could provide artifact-free measurements from possible optical interferences of biological tissues in emission intensity. Another example is that of a porphyrin-dimer-based molecular rotor with an emission spectrum that changes in response to the medium’s viscosity [174]. This porphyrin-dimer was used to probe intracellular viscosity changes due to prolonged irradiation in HeLa cells monitored by confocal fluorescence imaging. Myoglobin and polydopamine-coated gold nanoparticles assembled in a core-satellite motif were engineered as Raman nanoprobes for imaging reactive oxygen species in biological samples and living cells [175]. This example illustrates the versatility of porphyrin-based nano-assemblies to participate in different optical sensors besides imaging applications based on photoluminescence emission.

## 3. Outlook

The present review covering the topic of fluorescence of porphyrins and phthalocyanines intends to gather some experimental results obtained using different fluorescence techniques covering the interaction of these important molecules with different scaffolds to which the noncovalent association is favored within the context of self-assembly.

The large part of fluorescent data reported in the literature, as well as that obtained in our own group, was in aqueous solutions and in heterogenous media, but is still rather limited. Nevertheless, we believe it is of interest and representative of both the interest and impact on this field, which may be viewed as model systems with relevance toward the investigation of artificial photosynthesis.

The inclusion of the imaging microscopy section in this review, even though outside the self-assembly context, covers several examples of optical sensors, which integrate different types of quite elucidative “synthetic motifs”. In our opinion, the examples therein will certainly constitute, within the near future, an important research area that will expand and strengthen the domain of fluorescence spectroscopy.
